# Modelling pandemic behaviour using an economic multiplayer game

**DOI:** 10.1038/s41598-022-17642-w

**Published:** 2022-08-05

**Authors:** Simon T. van Baal, Lukasz Walasek, Jakob Hohwy

**Affiliations:** 1 Department of Psychology, University of Warwick, Coventry, UK; 2 Cognition and Philosophy Lab, Monash University, Melbourne, Australia; 3 Monash Centre of Consciousness and Contemplative Studies, Melbourne, Australia

**Keywords:** Disease prevention, Health care economics, Public health

## Abstract

During a pandemic, isolating oneself from the community limits viral transmission and helps avoid repeated societal lockdowns. This entails a social dilemma—either distance oneself from others for the benefit of the public good or free-ride and enjoy the benefits of freedom. It is not yet understood how the unfamiliar incentive structure and interpersonal context presented by a pandemic together modulate individuals’ approach to this social dilemma. In this preregistered study, we take a game-theoretical approach and investigate people’s decisions to self-isolate, using a novel iterated multiplayer game designed to capture the decision-making environment in the pandemic. To elucidate players’ thinking, we use a variation of the strategy method and elicit beliefs about how much others will self-isolate. Players tend to respond to social norms with abidance, rather than transgression; they resist the temptation to freeride when others are self-isolating. However, they deal with exponential growth poorly, as they only self-isolate sufficiently when lockdowns are imminent. Further, increased collective risk can motivate more self-isolation, even though the link between self-isolation and lockdowns is stochastic. Players underreport the influence of others’ choices on their own, and underestimate others’ self-isolation. We discuss implications for public health, and communication to the public.

## Introduction

Achieving high levels of protective behaviours during a major health crisis such as the COVID-19 pandemic has proven difficult in many countries. Adopting protective behaviours, like staying home when it is feasible to do so, carries substantial individual costs, whereas benefits apply to both the individual and the larger society^1^. Decisions on isolating oneself by staying home thus constitute a social dilemma^2–4^. Due to this conflict between interests of the individual and the collective, many countries had to enforce ‘lockdowns’ with enforceable stay-at-home orders as high disease prevalence put strain on healthcare systems.

It is not yet known if patterns of behaviour expected in non-pandemic settings also occur in the unusual incentive structure and social context of a pandemic, nor whether that unfamiliar setting modulates attempts to resolve the social dilemma in unexpected ways. Here we study how people make decisions about self-isolation during a pandemic scenario under the threat of lockdown, and look for areas of improvement for increasing the willingness to self-isolate and avoid future lockdowns. We are interested in how people react to two types of informational inputs: situational cues that signal the current viral transmission status and incentive structure changes, and social cues that reveal dominant behaviour patterns.

### Studying determinants of protective behaviour during a pandemic

We may try answering questions about the willingness to self-isolate through the use of cross-sectional surveys or longitudinal studies. There has been a proliferation of research in this area, which has brought many insights. However, drawing inferences on what drives behaviour from survey data is challenging considering the likely prevalence of recall bias^5^, social desirability bias^6–9^, potential confounds, and difficulty in dissociating causality from correlation. Likewise, using observational data on behaviour is often marred with privacy concerns, especially when it comes to personal information like someone’s health status.

Instead, we may turn to previous social psychology literature on how humans cooperate, and how they create and respond to social norms^10–12^; social norms are powerful drivers of behaviour, especially when people are unsure of the right course of action, thus predicting high levels of conformity in a pandemic. Although it is also clear that while some act in accordance with social norms, many act in their self-interest^13^. We may also conjecture that because people are suggested to suffer from exponential growth bias^14^ (people are, for example, not good at predicting the next number in an exponentially growing sequence of numbers, but see^15^), they may be unwilling to make costly sacrifices for public health when the caseload is still low. However, the COVID-19 pandemic has created a novel, unfamiliar situation for individuals and groups, and it is not clear how those tendencies *ceteris paribus* apply to the unique and unprecedented realities of the pandemic.

Economic games are well-suited to address questions about the determinants of willingness to self-isolate, and the effects of environmental changes because of the controlled setting in which behaviour is studied. At the same time, simply drawing inferences from existing context-free economic games carries the risk of failing to account for the unusual but ubiquitous circumstances of the pandemic. For instance, the stochastic link between behaviour (e.g., self-isolation) and the consequences to that behaviour (e.g., lockdowns) might introduce different choice patterns because it leaves more room for the influence of social norms and beliefs about others’ actions. In addition, the initial exponential stage of disease growth, paired with the influence of superspreader events, makes individuals’ decisions more influential than in traditional multiplayer games such as the public goods game. To deal with generalisability issues, the literature on economic games has broadened and is now rich with studies tailoring games to specific contexts, such as public goods provision in competing teams^16^, contributions to combat climate change^17^, building a dam to prevent flooding when there are private solutions available^18^. We contribute to this line of research with a game specifically designed to study the self-isolating behaviours in a pandemic.

In this study, we aim to investigate people’s willingness to self-isolate, through the use of a novel context-specific economic game. The game mimics the incentive structure of the pandemic, where self-isolation is costly but benefits the collective through decreased frequency or avoidance of lockdowns. The virus spreads exponentially, depending on whether infected players and non-infected players self-isolate. Naturally, our game provides a simplified view of the realities of an infectious disease outbreak; two notable simplifications are that individuals do not incur the costs of being infected and that participants do not know when they are infected. This allows us to focus on players’ responses to collective costs, rather than the individual cost of the disease.

We constructed this game to find out whether the incentive structure is sufficient to produce behaviour patterns that fit with the observations in the COVID-19 pandemic, and to ascertain how dynamically changing situational and social cues influence this behaviour. Important behaviour patterns that we tried to reproduce include the fact that people have been unable to avoid high disease prevalence and lockdowns without intervention, with nevertheless sustained willingness to self-isolate over time (as witnessed with the lack of “behavioural fatigue”^19^). To gain deeper insight into people’s responses to situational and social cues, the game uses a variation to the strategy method^20^, where participants indicate how much they would self-isolate in hypothetical scenarios and reveal their beliefs about what others will do in the next round.

The Self-Isolation Game presented here is thus related to, but contrasts with both existing context-free, classical games and games tailored to other specific scenarios. This game uses the advantages of economic games and applies them to the context of the pandemic to shed light on how individuals cooperate and behave to protect their interests as well as those of the broader social (public) good. Having participants operate under unfamiliar transmission dynamics, with a stochastic link between choices and outcomes, allows for a better understanding of what drives behaviour during infectious disease outbreaks and improves our ability to gauge the effect of manipulations in the environment.

### Phenomena of interest

The current gaps in knowledge on the motivating factors for people’s willingness to self-isolate that we investigate here, consist of the following four points. First, our game allows us to determine how people perceive and respond to descriptive social norms in their willingness to self-isolate, and whether they realise the influence these norms have. Descriptive social norms of high self-isolation levels could stimulate conformity^10^, which usually takes the form of conditional cooperation in economic games^21–23^, although descriptive social norms are less effective in regulatory contexts of prevention^24^, such as in a pandemic. Thus, descriptive social norms may promote norm transgression instead; people are tempted because when others are self-isolating, they think they can go out without getting or spreading the disease.

Second, with the use of our game, we assess whether people can deal with the exponential growth of viral transmission appropriately. People tend to underestimate how fast COVID-19 cases grow, imagining they grow linearly—known as exponential growth bias^25,26^—which may inhibit people from self-isolating effectively because it limits their capacity to see the danger in low case numbers. If there is a link between exponential growth bias and behaviour, then we should expect that people respond only to relatively high case numbers.

Third, insights from our paradigm can contribute to research on the reliability of behavioural self-report measures during a pandemic, including the potential interference of social desirability bias^6,8,9^. While the flaws in self-report data have been widely documented, studies using behavioural self-report measures are still overrepresented in the pandemic-behaviour literature. Considering that the unfamiliarity of the pandemic context may interact with response biases in new ways, it is important to gain more insight into how self-reports deviate from actual behaviour.

Lastly, we investigate whether increased costliness of the collective risk motivates more self-isolation behaviour when there is a stochastic link between behaviour and outcome (i.e., one individual’s action does not automatically translate to viral transmission or lockdown), and compare this to income maximising behaviour, as derived from simulations.

We conduct simulations to identify what a profit maximising player would do for various levels of others’ compliance, and different levels of disease prevalence. A profit maximising player would always defect (not self-isolate at all), regardless of others’ self-isolation levels, but the simulations reveal that defection becomes relatively more attractive when self-isolation levels in the group are high. Further, a profit maximiser would see no reason to increase their self-isolation when faced with increasing disease prevalence. Considering the substantial compliance with public health restrictions observed during the COVID-19 pandemic, we expect to find players adopting more cooperative strategies. Therefore, we aim to see if and how players’ behaviour deviates from this profit maximising strategy while addressing the four gaps in knowledge mentioned above.

### Predictions

We preregistered several predictions on people’s behaviour in the Self-Isolation Game, each of which speaks to the above phenomena of interest. For ease of presentation here, the ordering of the predictions differs from the ordering in the preregistration, as follows: H2, H3a, H3b in the present text are H3, H2i and H2ii in the preregistration, respectively.

Considering the game’s modelling of the exponential spread of the virus, the least costly way to avoid lockdowns would be to self-isolate to a high degree early on, when there are few infected players (for details, see Methods). We therefore predicted that (H1) players will be unable to deal with *exponential growth*, and self-isolate sufficiently only when it is too late—when there are several infected players in the group. That is, they do not account for exponential growth properly: they forfeit income even when it would be fairly inconsequential to defect because a lockdown is increasingly inevitable with more players being infected.

For the *accuracy of beliefs about others’ behaviour*, we predicted that (H2) players will believe that others will self-isolate more when there are more infected players in the group. In an exploratory analysis, we also assess whether participants suffer from *illusory superiority* (also called the better-than-average-effect, referring to the tendency to regard one’s own qualities and attributes as superior to others^27^), where they believe others will self-isolate less than they will themselves. We found this tendency in self-reports on staying home during a lockdown^28^. Illusory superiority is relevant in a disease outbreak context because it is known to hamper interpersonal adjustment, and it has been speculated to increase risk taking^27^.

Our game bears resemblances to the volunteer dilemma^29^ and the step-level public goods game^30^, where a sufficient number of players needs to cooperate to produce some binary public good (here: the lack of lockdowns). In contrast to games such as the prisoners dilemma, trust games, and ultimatum bargaining, cooperation and defection tend to coexist in the equilibrium. Therefore, we predicted that (H3a) players will self-isolate less when they believe that others will self-isolate more because defection is least consequential when others are self-isolating rigorously. We also predicted that (H3b) players will report that they would self-isolate less in hypothetical scenarios where others self-isolate more (i.e., we predict that norm transgression tendencies from H3a are reflected in their hypothetical responses).

We reasoned that people would exhibit social desirability bias, and thus we evaluate whether players’ *self-reports* in hypothetical scenarios indicate higher levels of self-isolation than what players *actually* choose during their incentivised trials; we predicted that (H4) players will indicate higher levels of self-isolation in hypothetical scenarios than in the subsequent actual (incentivised) trials.

Investigating whether a *collective risk* can motivate larger contributions even when there is a stochastic link between behaviour and outcomes, we predicted that (H5) costlier lockdowns lead to higher self-isolation levels. If lockdowns are costlier, then the benefit of self-isolation increases, and thus we should find that people respond to this by self-isolating more. But the probabilistic influence of self-isolation on disease prevalence may cause players to feel that whether a lockdown occurs or not is out of their control, which makes this worth investigating.

## Materials and methods

### Participants

The final sample consisted of 134 participants: 57 (42.5%) males, 76 (56.7%) females, and 1 (0.7%) indeterminate/intersex/unspecified with a mean age of 35.5 (*SD* = 12.0). Participants were required to be fluent in English, residents of the UK. Participants were excluded before completion of the experiment if they were diagnosed with dyslexia, dyspraxia, attention deficit hyperactivity disorder, or if they had trouble reading for any reason (including uncorrected abnormal vision).

The data collection was completed between 20 and 23 January 2021 via the Prolific online research participant database. At this stage in the COVID-19 pandemic, the UK was on the cusp of 100.000 COVID-related deaths, and two weeks earlier the National Health Service (NHS) England's national medical director urged people to physically distance because the NHS was under extreme pressure. The country was in lockdown without a clear end date. In addition, 3.07% of participants reported they had received a positive COVID-19 diagnosis.

The experiment was divided into 14 sessions with 11 participants each, for an initial total of 154 participants. The minimum group size after exclusions per session was eight. Any sessions that did not meet the cut-off were excluded from data analysis. 20 participants were excluded, most of whom dropped out while waiting for the experiment to start (19), 1 participant provided low effort data by missing too many responses. We departed from the preregistration here. We stated that we would discard data on a particular DV for participants who provided the same responses in 95% of the rounds for that DV. Seeing as there are legitimate reasons to respond the same each round, we did not go through with this exclusion procedure. Instead, if participants’ pages timed out in 30% of the rounds or more, their data was excluded.

Participants were paid £2.00, with a bonus of £0.20 for every 100 points (the endowment for each round). There were 40 rounds, so participants could accrue a maximum of £10. All participants provided informed consent as approved by the Monash University Human Research Ethics Committee (Project ID: 26499).

### Apparatus

This experiment was an online study, and participants were asked not to use mobile devices. No default options were used for any of the questions, and the options were always presented left to right in increasing order to avoid any confusion. The pages had timeout timers that would only appear when time was running out. This was done to streamline the experiment and make long waiting times unlikely while limiting the effect on the participants.

### Procedure

Participants began the study by completing a short questionnaire, stating their age, their sex, whether they had tested positive for COVID-19 in the past, and in which region within the UK they resided (i.e., England, Scotland, Wales, or Northern Ireland). Further, as an attention check, they were asked which city was not a city in the US, with the list including Tokyo, but with a subheading that read: “Regardless of the right answer, please select Chicago”. The attention check was not preregistered, but was added to flag submissions for more careful review. We could not find any reason to exclude the participants who failed the attention check, and found that excluding them would make little difference to the results (see Supplementary Materials for additional information, and a secondary analysis without these participants in the sample). Afterwards, they were presented with an introduction and instructions on how to play the game, which was created using oTree^31^.

Participants were made aware that a randomly selected player in the group was infected with COVID-19 and that they could spread it to other players in the group. They did not know who this ‘patient zero’ was, and were told that they could be patient zero themselves. Additionally, they would not know whether they were infected at any point, nor would they experience a decrease in income if they got infected. When six players were infected with the virus, the group would go into lockdown for two rounds, costing either 60 or 90 points per round, in the Low Cost and High Cost conditions, respectively. After a lockdown, a new patient zero was randomly selected. Players were notified that they were playing to get points and that to do this they could avoid lockdowns by reducing viral transmission through self-isolation (see instructions here https://osf.io/tr6y3/?view_only=aa314332c12c45548484ac084feed2f0).

**Figure 1:**
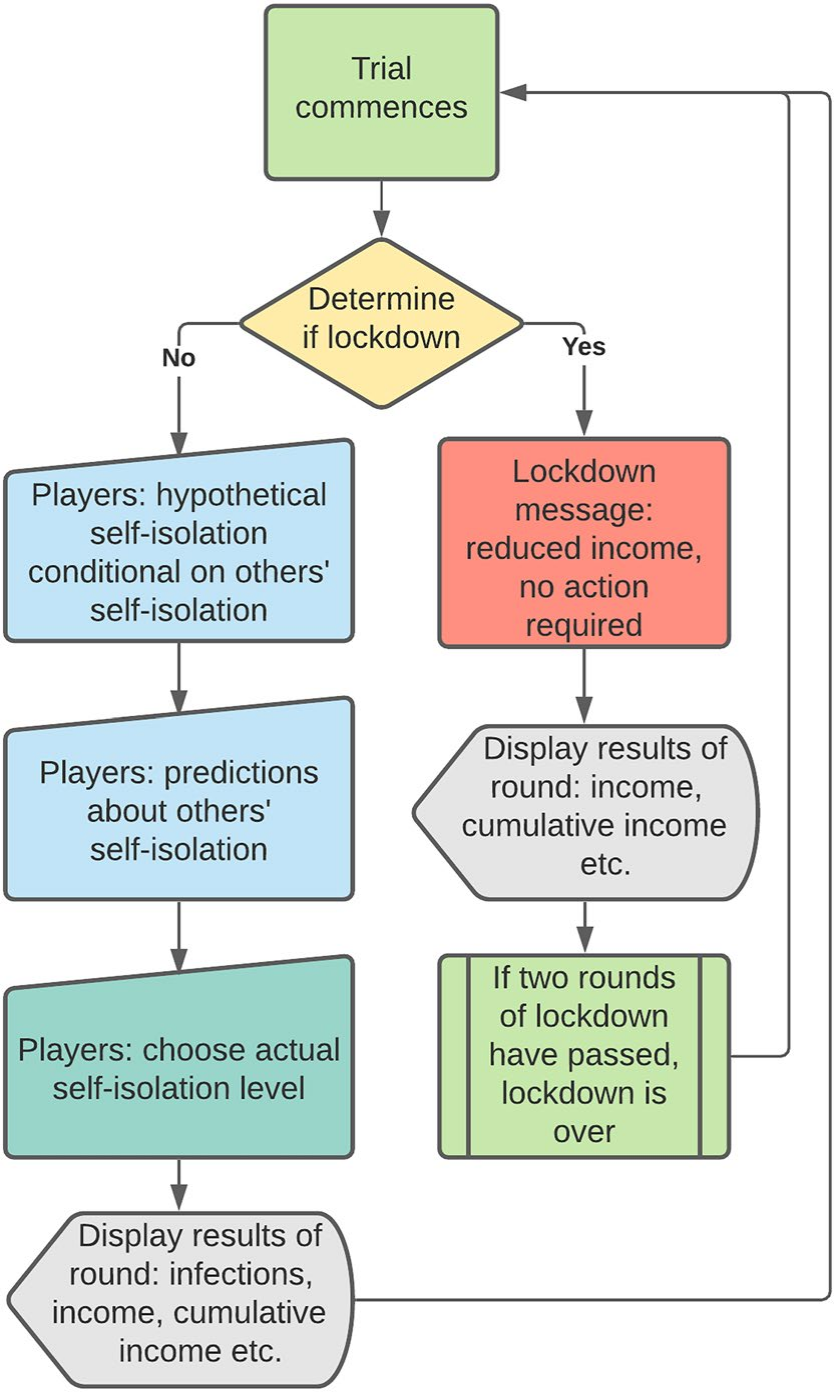
A trial-level schematic of the game from the players’ point of view.

In each round, the game required several responses from the participants; if one of the pages timed out before they responded, they were not paid for the round. First, we implemented a variation to the strategy method: to see how players would respond to different levels of self-isolation in the group, players were asked “If you knew what the average self-isolation level of the others in the group was in this round, how much would you self-isolate?”, with five sub-questions reading: “If the group self-isolation average were—[insert level]”. Thus, each of these sub-questions presents a different hypothetical scenario (hereafter: others’ self-isolation in scenario). For each sub-question, the response could be one of 5 self-isolation levels: not at all, slightly, moderately, stringently, or completely (hereafter: hypothetical self-isolation). Second, participants would provide their response to “On average, how much do you think others will self-isolate in this round?”, choosing one of the aforementioned options (hereafter: beliefs about others’ behaviour). Last, they answered: “How much do you actually want to self-isolate in this round?”, emphasising that this response would be incentivised (hereafter: incentivised self-isolation, see below). See Fig. 1 for the flow of the experiment from the participants’ point of view.

Participants’ endowment was 100 points in each round and they could sacrifice a part of their income by selecting one of the following options: sacrifice no points by choosing the self-isolation level “Not at all”, sacrifice 10% (i.e., 10 points) to slightly self-isolate, 20% to moderately self-isolate, 30% to stringently self-isolate, or 40% to completely self-isolate. Participants were not made aware of how much the self-isolation would decrease the chance of transmission (because this is also not the case in the real world).

**Figure 2:**
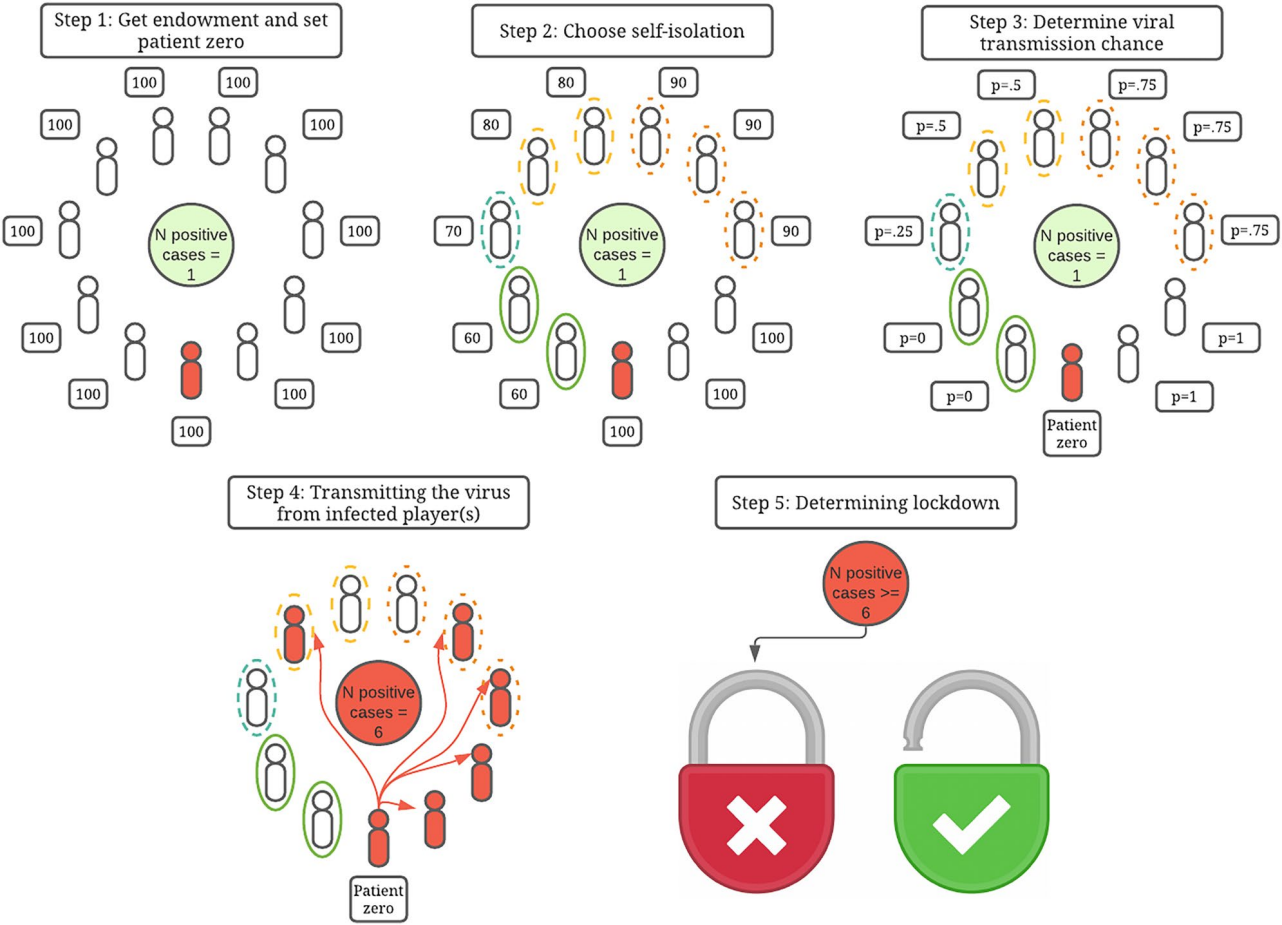
The costs and a demonstration of the viral transmission in the game. Participants all get 100 points of endowment in each (non-lockdown) round. They are then given a chance to choose their level of self-isolation to a maximum of 40% of their income, leaving minimally 60 points. This case depicts a scenario with only one infected player who does not self-isolate at all (at the bottom of the group, in red). Players who aren’t yet infected and do not self-isolate at all have 100% chance of getting infected, whereas players who self-isolate moderately (20% of endowment sacrificed) have 50% chance to get infected. The different self-isolation levels are indicated by differences in circles around the players (e.g., the green solid line is complete self-isolation (40% of endowment sacrificed), the dark orange dashed circle is slight self-isolation (10% of endowment)).

The transmission chance was determined through a stochastic process: if an infected player and a healthy player would not self-isolate at all in the same round, then the chance of transmission would be 100%, while if one of them would self-isolate moderately, this would be reduced to 50%. For example, if an infected player would self-isolate moderately, but a healthy player would not self-isolate at all, then the chance of the infected player transmitting the virus to the other player would be 50%. See Fig. 2 for the dynamics of transmission and the by-trial process.

Participants were all subjected to both lockdown cost conditions, and the order of presentation was counterbalanced. After 20 rounds in the initial lockdown cost condition, halfway through the experiment, either High Cost or Low Cost, participants were shown a message stating the new lockdown cost regime. The Low Cost condition was set to 60 points because it would equate incomes of groups where every member is completely self-isolating (i.e., for three rounds: 3*60 points), and not self-isolating at all (i.e., for three rounds: 100 points + 2*40 points in lockdown). The High Lockdown Cost condition was chosen to provide a strong contrast to this situation where not self-isolating resulted in efficiency loss; providing only 2/3 of the income of a fully self-isolating group (100 + 2*10). The range of outcomes for participants was, then, £4.98 (complete self-isolation and maximum number of lockdowns) and £10 (no self-isolation and no lockdowns).

After players entered their choices, they were presented with essential information regarding the results of that round. Specifically, they were shown: how much they had earned (i.e., endowment—self-isolation cost, or lockdown endowment); average self-isolation of others, as a percentage of complete self-isolation; how many infected players there were, and how many players there were in the group; their cumulative earnings in points. The presented information during the various participant inputs was held constant, and so participants were always shown how many infected players there were in the group, and what the cost of a lockdown would be if it were to occur after that round. For further details of the procedure, see Supplementary Materials.

### Analysis

#### Simulations

To compare participants’ behaviour to profit maximising strategies we simulated the income generated by adopting different strategies along the empirical boundaries of the game. We vary other players’ cooperativeness from complete defection (all other players select ‘no self-isolation’ in each round), to complete cooperation (all other players select ‘complete self-isolation’ in each round). We first simulated the game’s outcomes for the five possible *static* strategies, where the fictional player of interest maintains the same level of self-isolation throughout the game, among the changing environment of the other 10 players’ cooperativeness. For all strategies, income drops strongly as others become less cooperative. However, under both levels of lockdown cost, and the different levels of cooperativeness of the group, less self-isolation is more profitable.

In addition, we modelled the performance of *dynamic* strategies whereby players increase or decrease their level of self-isolation when infections rise. The fictional player of interest starts by choosing moderate self-isolation in every round but will either increase their self-isolation to complete self-isolation (‘Moderate to Complete’ strategy), or decrease it to no self-isolation (‘Moderate to None’ strategy) when there are more than two infections in the group. We compare this to the static ‘Moderate Cooperator’ strategy, where the player always chooses moderate self-isolation in each round.

#### Preregistered analyses

Based on the within-subjects design with only one manipulated variable (lockdown cost, with two levels), and various observed variables, we opted for a linear mixed modelling approach, which allowed for the controlling of the dependence between observations. There were three dependent variables (DV): incentivised self-isolation, hypothetical self-isolation, and beliefs about others’ behaviour. There were three other independent variables (IV) of interest: the cost of lockdown, the number of infections in the group, and the self-isolation of others in hypothetical scenarios. Other observed variables were used as control variables, these were: the round in which the observations were made and the average self-isolation of others in the previous round.

A linear mixed model (LMM) was used to analyse the effects of the number of infected players (H1) and beliefs about others’ self-isolation (H3a) on incentivised self-isolation. In this model, the DV was incentivised self-isolation, and the IVs were the number of infections, the self-isolation of others in the previous round, people’s beliefs about others’ self-isolation, and the round number. The model included participants as a random intercept and the round number as a random slope.

Another LMM was used to analyse the effects of the number of infections on beliefs about others' self-isolation (H2). In this model, the DV was beliefs about others’ self-isolation, the IVs were the round number, the number of infections, the self-isolation of others in the previous round. The model also included the participants’ IDs as the random intercept and the round number as the random slope. A third LMM was used to analyse the effects of others’ self-isolation in a scenario on self-reported hypothetical self-isolation (H3b). In this model, the DV was hypothetical self-isolation, and the IVs were the round number, the number of infections, the self-isolation of others in the previous round, and others’ self-isolation in the scenario. The model also included participants’ IDs as the random intercept and the round number as the random slope.

A Wilcoxon Signed Rank (WSR) test was used to measure the difference between hypothetical self-isolation and incentivised self-isolation (H4). Here we averaged self-isolation for each category (i.e., hypothetical or incentivised trials) per participant, such that each participant had one observation in each category. Only the hypothetical scenario was used that the participant believed would happen (e.g., when the participant indicated they believed others would choose moderate self-isolation levels, then that hypothetical self-isolation response would be used for this analysis). To measure the effect of the cost of lockdown on incentivised self-isolation (H5), we also used a WSR test. The WSR test was used in these instances because the tests are designed to only detect a main effect, disregarding any interactions.

Analyses were conducted using R^32^, mainly relying on the “afex” package^33^ and the “emmeans” package^34^. Results were considered significant based on a false discovery rate adjusted α of 0.05.

#### Exploratory analysis

One exploratory analysis was conducted to investigate whether participants consistently self-isolated more than they believed others would. This analysis consisted of a WSR, testing the difference between incentivised self-isolation (aggregated per player) and beliefs about others’ behaviour (aggregated per player).

The preregistration can be found ﻿here https://osf.io/xhf7w/?view_only=c3f6f12d380d414f8584e19b7a934294; the data, analysis code, and code for the experimental software are all publicly available on the Open Science Foundation website here https://osf.io/jcmwt/?view_only=d7b6fe0e5e53447ca871312b2b10f779.

### Ethical approval

This research was approved by the Monash University Human Research Ethics Committee (Project ID: 26499). The experiment was performed in accordance with relevant named guidelines and regulations. Informed consent was obtained from all participants and/or their legal guardians.

## Results

### Simulations

**Figure 3:**
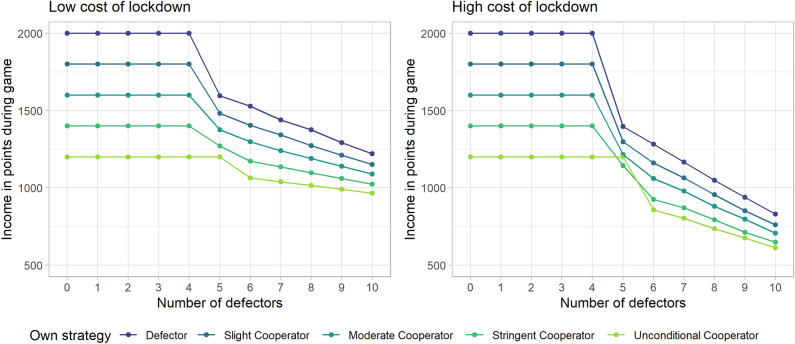
A comparison of average cumulative incomes achieved in simulations of the game through all five possible static strategies. The panel on the left represents the Low Lockdown Cost condition, wherein players earned 40 points per round in lockdown, whereas the panel on the right shows the condition wherein players earned 10 points per round in lockdown. The income earned through adopting each strategy (y-axis) is shown for a different number of defectors and unconditional cooperators in the group (x-axis); the left-most points describe groups where all other players choose ‘no self-isolation’ in every round (defectors), while the right-most points depict groups where all other players choose ‘complete self-isolation’ in every round (unconditional cooperators). The upper, purple lines represent the income one would receive when adopting a strategy of choosing ‘no self-isolation’ every round, and each line below shows the income received through a one increment increase in self-isolation.

The simulations show that the most profitable static strategy is to defect (by never self-isolating), regardless of what the rest of the group chooses to do. It is also clear that the less the others self-isolate, the less profitable each strategy becomes, but the ordering of the profitability of each strategy is largely maintained as the most cooperative strategies are the least profitable. The socially optimal outcomes are that everybody chooses ‘complete self-isolation’ in the High Lockdown Cost condition, and ‘stringent self-isolation’ in the Low Lockdown Cost condition. There is also a steep drop in profitability when there are 6 or more players in the group who are not self-isolating at all. This is because lockdowns will happen when six players are infected, affecting players’ endowments. See Fig. 3 for a depiction of the cumulative income attained by players per strategy for varying levels of cooperativeness of the others in the group.

**Figure 4:**
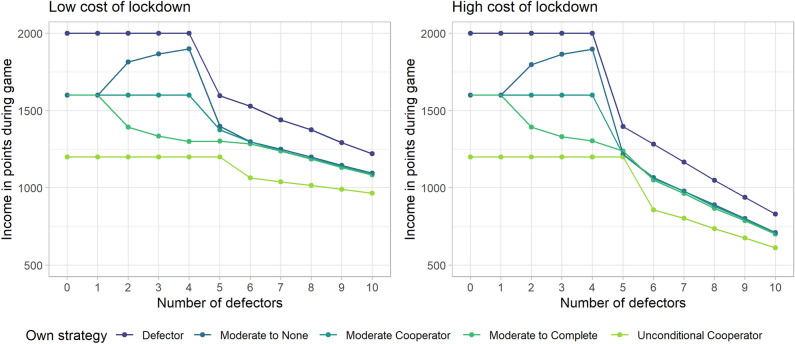
A comparison between dynamic strategies and static strategies. Two dynamic strategies are depicted, namely ‘Moderate to None’ and ‘Moderate to Complete’, in these strategies, participants choose ‘moderate self-isolation’ if there are only one or two infected players in the group. When there are more infections in the group, players who adopt the Moderate to None strategy always choose ‘no self-isolation’ whereas those adopting the Moderate to Complete strategy then choose ‘complete self-isolation’. The other three strategies are static strategies, where defectors always choose ‘no self-isolation’, moderate cooperators choose ‘moderate self-isolation’, and unconditional cooperators choose ‘complete self-isolation’.

When comparing dynamic strategies, where players start with one of the static strategies but adjust their self-isolation level when there are 2 or more infections, we found that decreasing self-isolation when cases increase is strictly dominant over a strategy of increasing self-isolation as well as holding the same strategy. This is especially true when there are relatively few defectors in the group, while producing the same amount of income for < 2 defectors and for > 5 defectors. However, a complete defection strategy is still strictly dominant. See Fig. 4 for a comparison between these dynamic strategies with some of the static strategies mentioned before.

### Experimental results

Participants maintained high levels of self-isolation throughout the experiment (see Figure S1 in supplementary materials for average levels of self-isolation per round). Female participants on average sacrificed more of their income to self-isolate (*M* = 23.43) than males (*M* = 21.63), and there was no apparent association between age and self-isolation.

**Figure 5:**
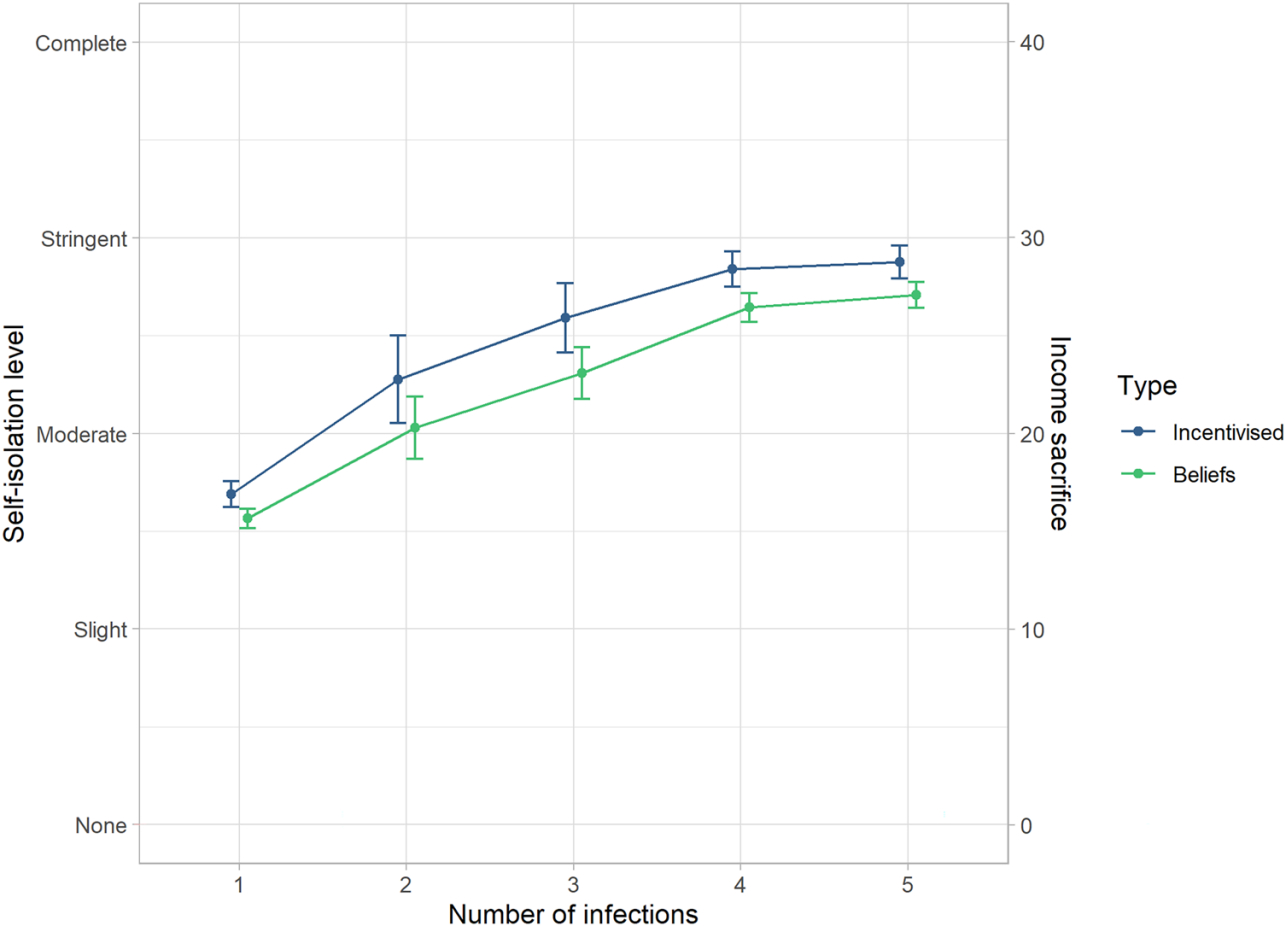
A visualisation of the effects of the number of infections in the group (x-axis) on incentivised self-isolation (in blue) and beliefs about others’ self-isolation (in green). The error bars represent 95% confidence intervals.

Our results show that H1 and H2 were supported. First, there was a significant effect of the number of infections on both incentivised self-isolation, *b* = 1.345, *SE* = 0.115, *t*(3317) = 11.684, *p* < 0.0001, and on players’ beliefs about others’ self-isolation, *b* = 2.833, *SE* = 0.105, *t*(3300) = 27.106, *p* < 0.0001. This means that the more infections there were in the group, the more they would self-isolate, and that players (correctly) believed that others would do the same. However, the exploratory analysis on illusory superiority showed that beliefs about others’ self-isolation (*Mdn* = 20.84) were lower than players’ own incentivised self-isolation (*Mdn* = 21.94), *z* = 2.198, *p* = 0.02. See Fig. 5 for the influence of the number of infections on self-isolation and beliefs about others, and Table 1 for a summary of the output of the three regressions. This means that players believed others’ self-isolation would be lower than their own self-isolation.

**Table 1:** A regression table showing the effects of various predictors on incentivised self-isolation, beliefs about self-isolation of others, and hypothetical self-isolation.

	Dependent variable		
	Incentivised self-isolation (1)	Beliefs about others (2)	Hypothetical self-isolation (3)
**Regression results H1–H3**			
Round number	0.022 (0.025)	− 0.014 (0.023)	0.007 (0.022)
Number of infections	1.345*** (0.115)	2.833*** (0.105)	1.803*** (0.063)
Others in previous round	0.009 (0.007)	0.009 (0.007)	0.007* (0.004)
Beliefs about others	0.475*** (0.017)		
Self-isolation of others in scenario			0.108*** (0.005)
Constant	22.328*** (0.698)	20.776*** (0.556)	22.803*** (0.770)
Log likelihood	− 12,091.020	− 12,064.220	−63,638.690

*p < 0.1; **p < 0.05; ***p < 0.01.

**Figure 6:**
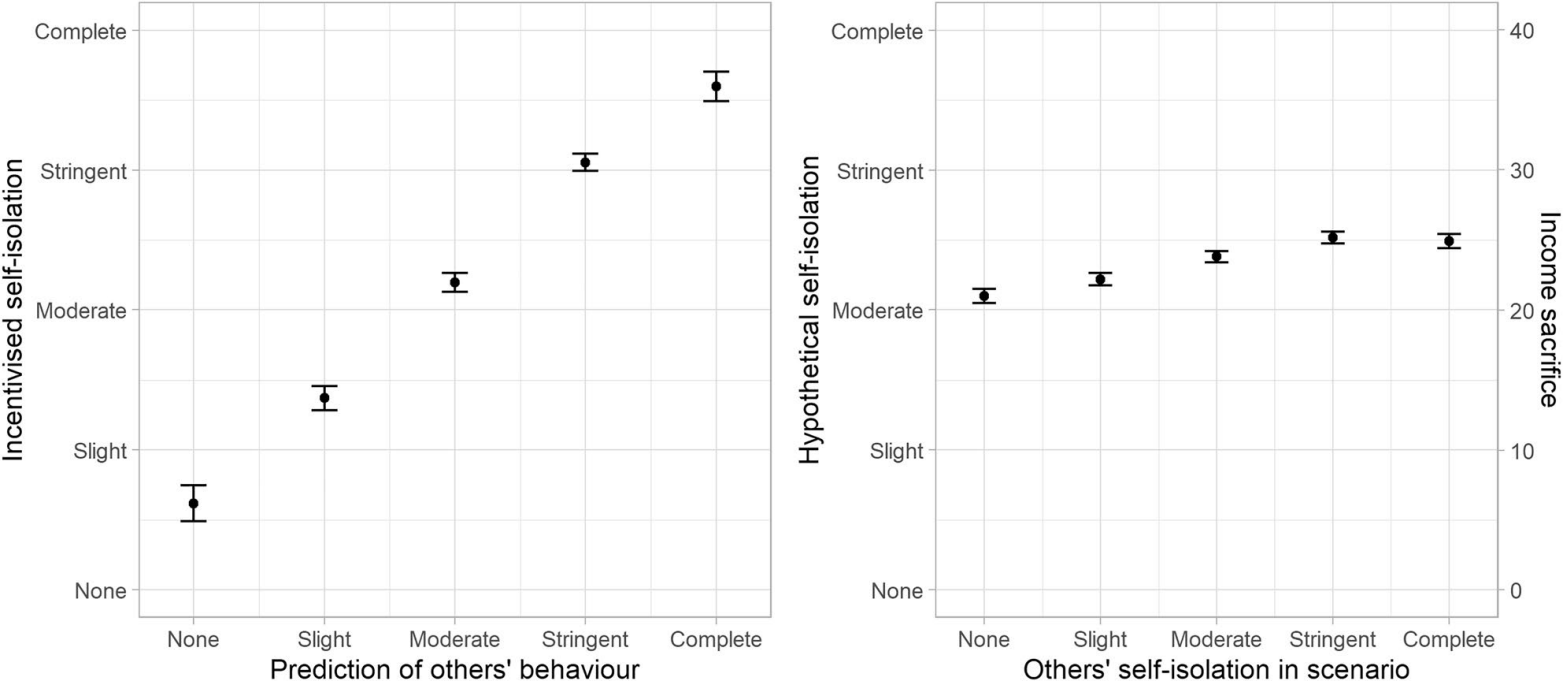
The effects of a player’s beliefs about other players’ self-isolation (x-axis in the left panel) behaviour on their incentivised self-isolation (y-axis in the left panel), and the effects of the self-isolation of others in each hypothetical scenario (x-axis in the right panel) on self-isolation levels (y-axis in the right panel). The error bars represent 95% confidence intervals.

We found an effect of descriptive social norms in the opposite direction of H3a: there was a significant effect of beliefs about others’ self-isolation on incentivised self-isolation, *b* = 0.475, *SE* = 0.017, *t*(3420) = 27.745, *p* < 0.0001. When participants believed that others would choose high levels of self-isolation, they chose higher levels of self-isolation themselves. Further, the results concerning H3a were mirrored in hypothetical scenarios (H3b). Participants reported they would self-isolate more in hypothetical scenarios where others were also self-isolating more, *b* = 0.108, *SE* = 0.005, *t*(16,810) = 20.470, *p* < 0.0001, but this effect was markedly smaller. See Fig. 6 for the influence of beliefs about others’ behaviour on incentivised self-isolation, compared to the effect of others’ self-isolation in a hypothetical scenario. This figure is relevant to evaluating what information about others motivates players’ behaviour.

Turning to the differences between self-reports and incentivised choices, we found no evidence for H4; self-isolation levels in hypothetical scenarios (*Mdn* = 22.48) were not significantly higher than incentivised self-isolation (*Mdn* = 22.13), *z* = 0.572, *p* = 0.28.

**Figure 7:**
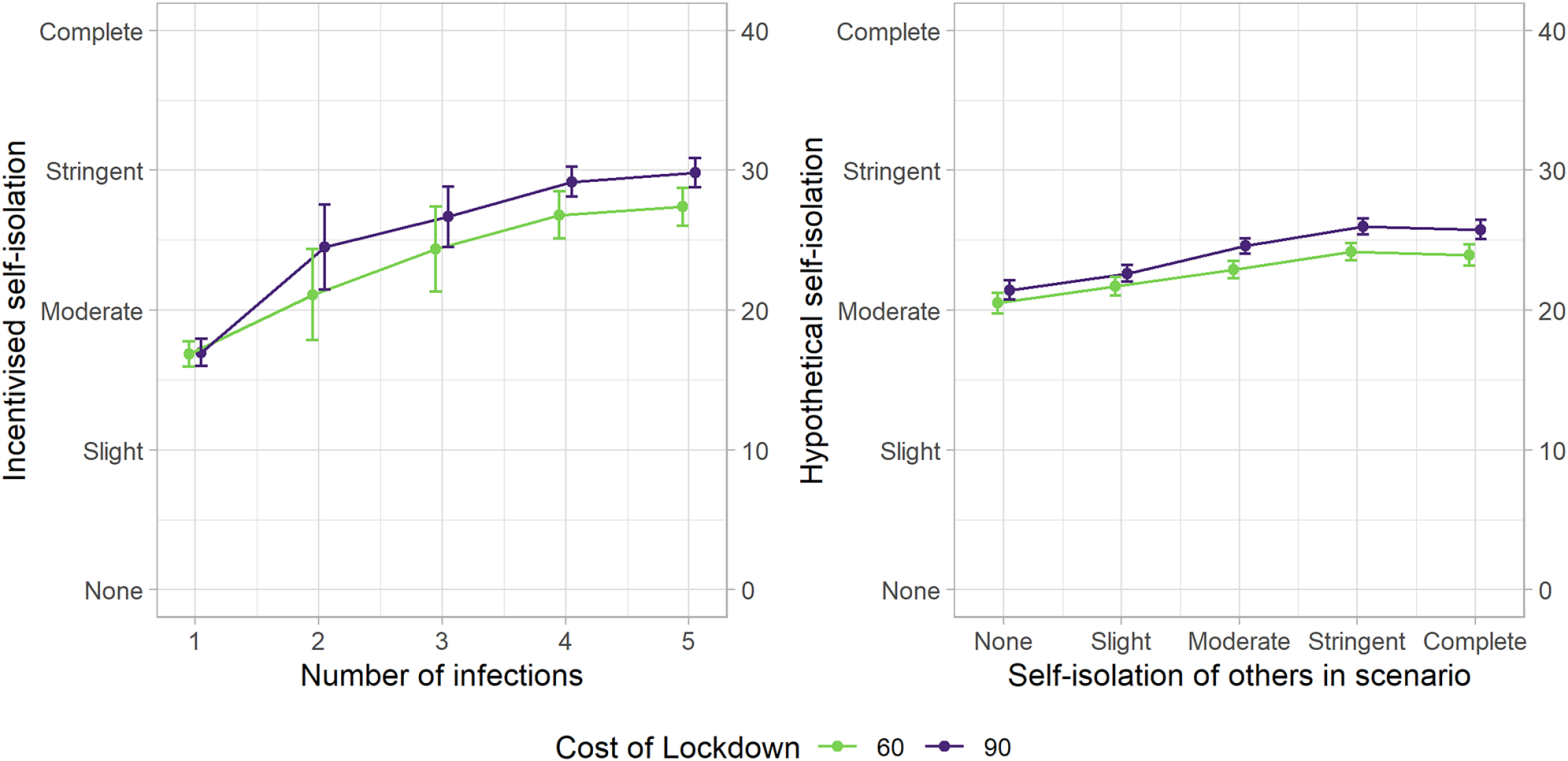
The effect of the cost of lockdown (60 points per trial in green, 90 points per trial in purple) on incentivised self-isolation levels (y-axis, left panel), parsed by the number of infections in the group (x-axis). In the right panel, a line plot of the interaction effect between the self-isolation of others in each hypothetical scenario (x-axis) and the cost of lockdown, on self-isolation (y-axis). The error bars represent 95% confidence intervals.

H5 was supported, however. There was a significant effect of the cost of lockdown on incentivised self-isolation. Incentivised self-isolation was lower in the Low Lockdown Cost condition (*Mdn* = 20.00) than in the High Lockdown Cost condition (*Mdn* = 23.66), *z* = 3.667, *p* < 0.001. This means that people self-isolated more when the cost of lock-down was high. See Fig. 7 for the interaction between the cost of lockdown and the number of infections on incentivised self-isolation.

## Discussion

The Self-Isolation Game we present in this study was designed to capture key properties of the self-isolation decisions that individuals face during an infectious disease crisis, such as the COVID-19 pandemic. The results were as follows: players only tended to self-isolate more as there were more infections in the group, when lockdowns were practically unavoidable. Players systematically underestimate other people’s willingness to self-isolate compared to their own—displaying illusory superiority. They also tend to respond to social norms with norm abidance, rather than norm transgression, even though decisions were private. Players’ willingness to self-isolate was affected by the cost of lockdown, but only in the second block. We discuss these results in detail below.

Players maintain relatively high levels of cooperation (i.e., self-isolation) throughout the game. In many other repeated games, contributions normally dwindle^35–37^, whereas in the Self-Isolation Game, players do not tend to decrease their self-isolation, even though they garner no direct benefits from their input. This implies that the incentive structure and context produce an environment where people remain motivated to cooperate, more so than in a classic repeated public goods game, but mimicking the absence of ‘behavioural fatigue’^19^ in the general public during the pandemic (see Figure S1 for the development of players’ average self-isolation levels throughout the game). One other explanation for the continued high levels of self-isolation may be that players receive information on the average contributions of the others in the group, rather than about each individual player’s contributions. The way information is presented is particularly relevant during the pandemic because prominent defectors (such as vocal anti-vaccination advocates) can instil feelings of helplessness and encourage non-adherence to guidelines^38^. Collective risk social dilemma games, which also bear significant similarities to the current game, can produce high levels of cooperation under the right conditions^17,39^. These conditions appear to be met with the current parameters of the game, even though the relationship between self-isolation and lockdowns is stochastic, and there is minimal efficiency gain of self-isolation when the cost of lockdown is low.

We predicted that (H1) participants’ self-isolation would increase as a function of disease prevalence. We reasoned that people would underestimate the importance of early intervention to stop the spread of the virus; that they would increase their self-isolation only when lockdowns were nearly inevitable. This prediction was supported, as participants persistently displayed relatively low self-isolation when there was only one case in the group, but ramped up their self-isolation gradually as disease prevalence rose, reaching maximum levels when lockdowns were already imminent (see Fig. 5). This result relates to a recent study reporting that COVID-conscious people were focused on the current disease prevalence numbers (rather than their possible contribution to a rise in those numbers)^40^. It is significant because if their goal was to avoid lockdowns, then maintaining a high level of self-isolation throughout would be a superior strategy. In addition, our simulations show that such a strategy is weakly dominated as a profit maximising strategy because players could earn more income by defecting after cases increased.

It thus appears that players are unable to deal with exponential growth appropriately, in a pattern that is consistent with exponential growth bias^25^. This pattern could also have arisen due to players initially exploring the consequences of low self-isolation levels, and subsequently responding to a rise in infections. Either way, rather than players ultimately overcoming this underestimation of the importance of self-isolating early, this pattern then gets ingrained, possibly because it turns into a descriptive social norm. This implies that waiting with the implementation of public health measures until popular support for those measures is attained may cause an ineffective, slow response. Arguably, this is one of the successes in many countries’ policy responses to the pandemic; the first containment measures were usually introduced within days or weeks after the first case was detected^41^.

Players believed (correctly) that other players would self-isolate more when the number of infected players increased, and thus H2 was supported. But, their beliefs about others’ self-isolation levels were systematically lower than their own self-isolation (if beliefs were accurate, these would be equal on average), see Fig. 5. This suggests that players suffered from illusory superiority; they tended to believe others would self-isolate less than they did themselves, a pattern we also found in another study^28^. However, it is more surprising in this context because players have complete knowledge about average self-isolation in previous rounds by other players. The type of illusory superiority we found in this study is also surprising because it goes against previous findings on the *holier-than-thou* effect where people overestimate themselves, but do not underestimate others^42^.

Illusory superiority is positively correlated with wellbeing and self-esteem and negatively correlated with depression^43^. Thus, it might be helpful to people to think they are more willing to adopt protective behaviour, such as self-isolation, than others; it makes them feel like they are good citizens who are contributing to the public good. Illusory superiority also potentially has negative implications for the management of a pandemic because their underestimation of other people’s compliance means that people may be more pessimistic about the probability of successful outbreak management. That is, when people underestimate the likelihood that others adhere to public health guidelines, they will infer that the measures are likely to fail. This may increase other forms of social distrust and non-compliance. In addition, although the link has seemingly not been explicitly tested, it has been proposed that illusory superiority may encourage people to engage in moral licensing^44,45^. That is to say that people may believe they have been behaving relatively morally (because they believe others are uncompliant), making it appear more permissible to transgress^46^.

The influence of social norms was the opposite of our predictions (H3a, H3b): we hypothesised that participants would be tempted to take advantage of others’ higher compliance and self-isolate less because free-riding is less likely to be consequential when others self-isolate more. The simulations show that the extra income that can be earned by defecting is much higher when others are diligent self-isolators, which is similar to the incentive structure in classic games such as the trust game^47^. However, players’ self-isolation was highly correlated with their beliefs about others’ willingness to self-isolate, favouring an explanation in accordance with the literature on social norms^48^ (See Fig. 4), which predicts that people behave per the descriptive social norm especially when they are uncertain about the correct policy^49^, but also that cooperative behaviour tends to cascade through social networks^50^. Another possibility is that the context of the pandemic changed how the norms are perceived, turning the behaviour of others into a prescriptive, rather than a descriptive, norm^11^. This is not to say that freerider behaviour does not exist in the pandemic, but it provides grounds to believe that the urge to follow social norms may be strong in this type of context.

The cooperation pattern we observed also relates to the literature on conditional cooperation in public goods games, a well-studied phenomenon^21–23^, but the difference here is that the stochastic nature of infections leaves room for sustained conditional cooperation throughout the repeated game. It has also been suggested that many people may not perceive behaviour in the pandemic as a social dilemma, and that general levels of trust and cooperation (i.e., beliefs and behaviours not specifically related to pandemic behaviour) are not a good predictor of intentions to adopt protective behaviours^51^. Thus, managing beliefs about descriptive social norms on willingness to socially distance and stay home could be a powerful communication tool for governments, but more research needs to be done on this topic and how it relates to different domains of behaviour during a pandemic.

We predicted that players would indicate more willingness to self-isolate in hypothetical scenarios than in incentivised trials (H4), as one would expect social desirability bias to work in this direction; people report that they would self-isolate a lot in a certain scenario (often a socially desirable answer), but might not do so. This hypothesis was not supported, closely aligning with the results of Larsen et al.^9^ and Gollwitzer et al.^8^, who also found no evidence of overreporting willingness to comply with government guidelines. The social desirability bias measurement in this experiment was stringent, however, because unlike in many social desirability bias studies, the self-report measurement was not retrospective (leaving no room for recall bias)—self-reports were temporally proximate to the actual behaviour.

Although, there was a downward bias in the extent to which they indicated their decisions were influenced by the descriptive social norm (see the difference between the left and right panels of Fig. 5). Tuncgenc et al.^52^ found that people’s decisions in pandemic contexts are highly influenced by close peers. Underestimating or being unaware of that influence would be particularly harmful in a pandemic context because people (‘self-isolation role models’) could use that information to influence their peers by setting a good example, which could improve their own outcomes (e.g., by avoiding a societal lockdown). It may thus be risky to trust people’s insights on what influences their decisions in self-report studies, and this is brought out in an experiment like our game that contrasts a type of self-report with actual incentivised behaviour.

We predicted that (H5) the cost of a lockdown would affect the level of self-isolation players chose in the game because people may be sensitive to the magnitude of the collective risk. This prediction was supported, but it was driven mostly by the second half of the game (after the cost of lockdown was changed). Players increased their self-isolation when they transitioned from the Low Cost to the High Cost condition, and vice versa, while their self-isolation levels in the first half of the experiment were comparable over the two levels of lockdown cost (see Figure S1). Therefore, it is likely that this is a framing effect; people think they need to self-isolate more when the collective cost of not doing so increases. This reinforces the importance of thoughtful management of the public’s perception of the collective risks to the community.

### Limitations and future directions

The experimental design left out any adverse effects of contracting the virus. The current paper is meant to not only stand on its own, but also serve as a template for how people’s behaviour under various payoff structures in future pandemics could be studied, and thus parsimony of the model was an important consideration. Omitting personal costs served to mimic the payoff structure for a key demographic that is difficult to motivate to adhere to public health guidelines: those who believe that catching the virus will not have an impact on them (i.e., the ‘infection indifferent’)^40^. It also served to ensure that we could capture key features of decision making during the pandemic, namely, how people respond to the risks and costs to the collective, and not whether they respond to their individual cost function. Nonetheless, gauging the impact of different individual costs associated with the virus on behaviour would be a meaningful addition to the current study because it could interact with the collective risk in various ways.

Another issue is that some of the findings in this study will be dependent on parameterization, the country of residence of the sample, or the framing, and teasing these influences apart is challenging. Changing the parameters of the game, the framing, and the sample will likely change the relationships that we found in this paper: for example, if we introduce inequality in endowments, some group members might be unresponsive to changes in the number of infections; if we change the framing of the game to remove all mentions of the pandemic, the baseline willingness to self-isolate may be lower; different cultures might handle information about other group members differently. Thus, for future research it is important that the rest of the realistic parameter space, the influence of different cultures and nations, and neutral (non-pandemic) framing, be explored to compare to the original findings.

For some findings, though, it seems unlikely that they would be affected by these manipulations. Illusory superiority would likely remain a feature of behaviour during the pandemic we also recorded it in a self-report study^28^, and because the game already provides participants with average compliance information. Similarly, we deem it likely to be a robust feature of human behaviour during the pandemic that people’s compliance with, and support for containment measures may accumulate slower than would normally be optimal from a public health perspective. That is, people will usually respond to new outbreaks (or perhaps ‘threats’ in neutral framing) when the pathogen has already taken hold in the community because only then will it become clear that it is a ‘problem’ and not an overreaction to try to contain it.

The group size in the experiment was kept at eleven participants, even in case of dropouts. Participants who dropped out were marked as complete self-isolators during the game, and therefore the self-isolation rigour in the group was artificially inflated (although these observations were dropped from analysis). This means that information presented about others’ self-isolation rigour was often exaggerated slightly (19 dropouts over 14 sessions led to an average ~ 6% overestimation of others’ compliance). With this in mind, the illusory superiority found here is even more striking because, given that others’ self-isolation was slightly inflated in this experiment, one would expect that players believe others self-isolate more than they do themselves. And even though we see no reason that the other findings of this experiment would change through allowing the number of players in the group to decrease after dropouts, further study should investigate any differences that would occur.

Further, the experimental software would reset and switch to a new patient zero if there was no transmission for three consecutive rounds. This modelled effective management of outbreaks and was inspired by the situation in countries where there was no community transmission, such as New Zealand (at the time of the study being conducted). Namely, if there is one positive case, and they do not spread the virus, then they will no longer be infectious after a while and the risk they pose to the community dissipates. In this scenario, the next possibility for a new chain of infection would be a different patient zero (e.g., from international travel). However, in all other scenarios, players would not similarly lose their infectiousness after three rounds, which would be more realistic, but we decided not to include this to preserve the parsimony of the model. Further study could look at how people’s behaviour changes if they know that they are infected and if they know when they will no longer be infected.

## Conclusion

The Self-Isolation Game shows several important behavioural tendencies in a disease transmission suppression setting that would have been difficult to investigate with classical games or survey-based research. We found that people exhibit illusory superiority, they can be motivated to sacrifice through a collective risk, and they tend to follow social norms even when doing so is disadvantageous for themselves and the group.

Policymakers should be aware of the effect that perceived descriptive social norms have on people’s willingness to self-isolate. Creating the perception of social norms around willingness to maintain physical distance and to stay home may alleviate the need to impose restrictions. Careful framing of the high cost of a lockdown may also induce more willingness to adopt protective behaviours.

Waiting for public support to impose measures against viral transmission may also be ill-advised; people are late to respond to growing outbreaks, which is why policy interventions may need to be introduced before the public perceive the threat as serious. Finally, people should be aware overestimating the rigour of one’s own protective behaviours compared to others is common, and use this to promote understanding and compassion for each other during difficult times.

## Supplementary Information

Supplementary Information.

## Data Availability

All data are available here, together with the analysis code, and the code for the experimental software.
