# Supplementary material for: Modelling pandemic behaviour using an economic multiplayer game
Source: Sci Rep. 2022 Aug 5;12:13466. doi: 10.1038/s41598-022-17642-w (PMC9355951; doi:10.1038/s41598-022-17642-w)

Supplementary Materials

Here we report details of the experimental procedure that will be beneficial for the replicability of the study, as well as the results of the secondary analysis that checks the robustness of the results against excluding all participants who did not pass the attention check.

# Methods

Participants were not made aware of how many rounds they had completed, and were told the experiment would be anywhere from 30-50 rounds (the sessions were always 40 rounds), in order to prevent any ‘last round effects’, meaning to prevent participants from returning to the Nash Equilibrium of the stage game (a one-round equivalent), which is to not self-isolate at all. This is the Nash Equilibrium in this particular setup because lockdowns (i.e., endowment deductions) follow in the next round, which is not possible in a stage game.

Participants were required to provide their choices before pages timed out, otherwise they would not be paid for that round. If participants did not submit their answers on time, the experimental software would mark their answer as complete self-isolation, so as not to negatively impact other players in the group (this happened infrequently, on average .32 times per participant after exclusions). This was done to ensure data quality. Participants were then able to progress to the results section, and subsequently to the next round.

We also chose to maintain the 11-player group size, even if there were dropouts, so as not to confuse the participants and change the dynamics of the experiment too much. In order to do this, dropouts had to be treated as complete self-isolators (this way the others would not be adversely affected by their absence). As a result, 19 dropouts over 14 sessions led to a minor overestimation (average ~6%) of others’ compliance.

The experiment would also automatically ‘reset’ and randomly select a new patient zero if none of the players transmitted the virus to anyone else for 3 rounds. This resetting modelled people losing infectiousness after a while, and it meant that the players had successfully managed a chain of transmission by self-isolating enough. However, this is not part of the results as it is not particularly informative in itself, given that this is highly dependent on patient zero’s self-isolation rigour. This happened 1.93 times per session on average (maximum 3 times).

Participants were shown a ‘lockdown message’ during each lockdown round, specifying how costly the lockdown would be (either 60 or 90 points), that it would last two rounds, and that they would not need to make any of the decisions they normally would. The ambiguous effect of the cost of lockdown can be seen in Figure S1. Participants start at a similar level of self-isolation until after the cost of lockdown is changed, at which points the two groups diverge—presumably because they infer increasing (decreasing) their self-isolation in response to an increase (decrease) in the cost of lockdown is the correct response.

# Results

For this experiment, the sample was split into two groups: those who were subjected to the high lockdown cost first and the low lockdown cost after, and those who were subjected to the reverse order. To show the effect of the cost of lockdown on participants’ behaviour, accounting for order effects, we plot the average contribution of participants per round in each group (see Figure S1).


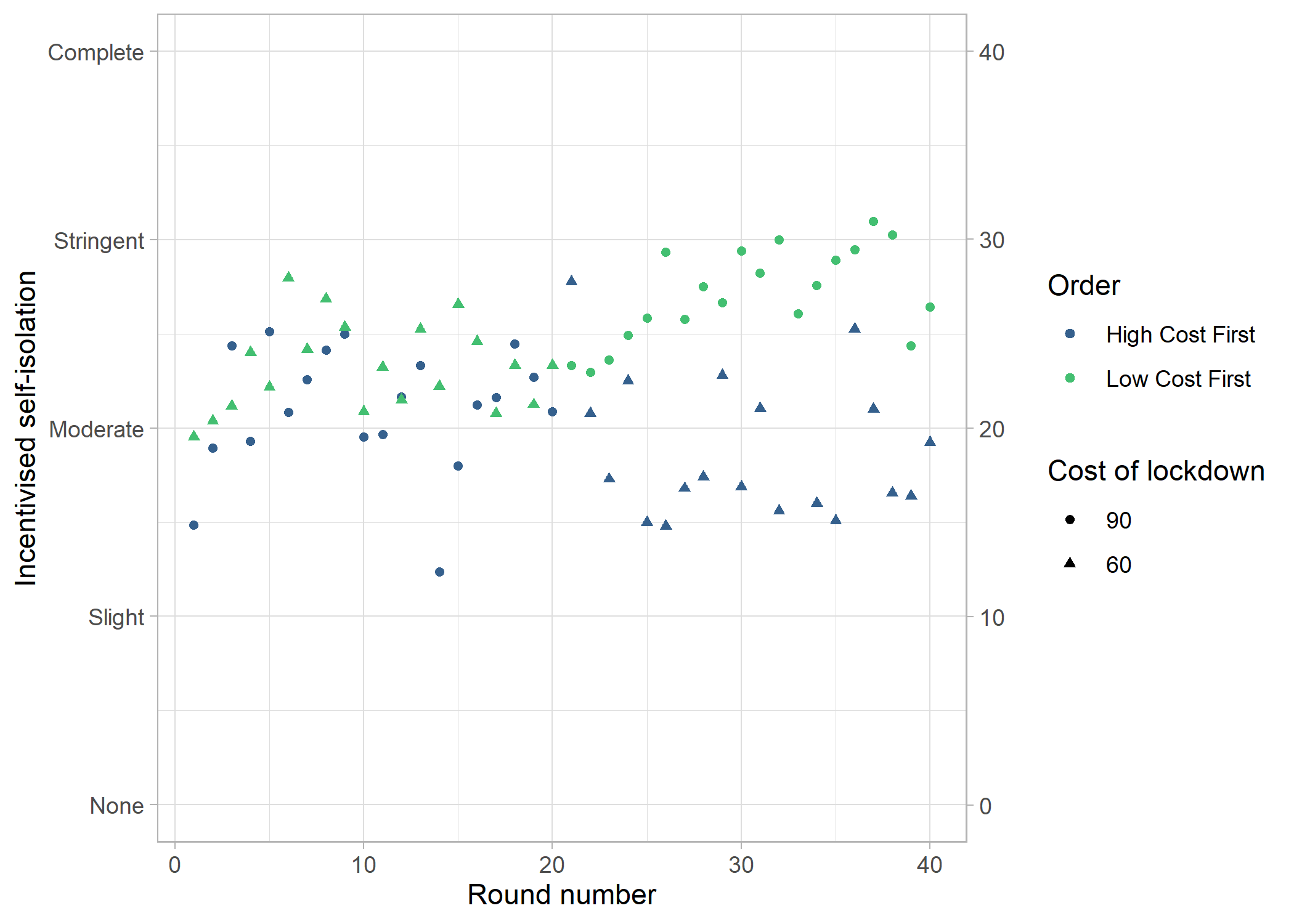


Figure S1. The average self-isolation levels throughout the game. Each point represents the average self-isolation (y-axis) of all groups during that round (x-axis) in one of the two orders in which the lockdown cost was changed, provided they were not in lockdown. The colour of the points is determined by the order in which the cost of lockdown was administered (blue is when the high lockdown cost condition was first, and green is when the low lockdown cost condition was first). The shape is determined by the cost of lockdown in that round (circles for high cost of lockdown, triangles for low cost of lockdown).

## Secondary Analysis

In the original analysis we did not exclude those who failed the attention check. There were no signs that data quality was compromised for these participants, nor was there any difference in the statistical test results. For transparency, we reanalysed the data without those participants to show that the results are robust to their exclusion.

Just as in the primary analysis, H1 and H2 are supported, and we observed an effect in the opposite of the predicted direction for H3a and H3b. See Table S1 for the regression results pertaining to H1, H2, and H3a,b. The results for H4 and H5 were also mirrored in the secondary analysis: H4 was not supported, *z* = .695, *p* = .24, but H5 was supported, *z* = 3.862, *p* < .0001. Furthermore, we found that participants also exhibited illusory superiority in this reduced sample, *z* = 2.245, *p* = .01.

Table S1. Regression results for the secondary analysis.


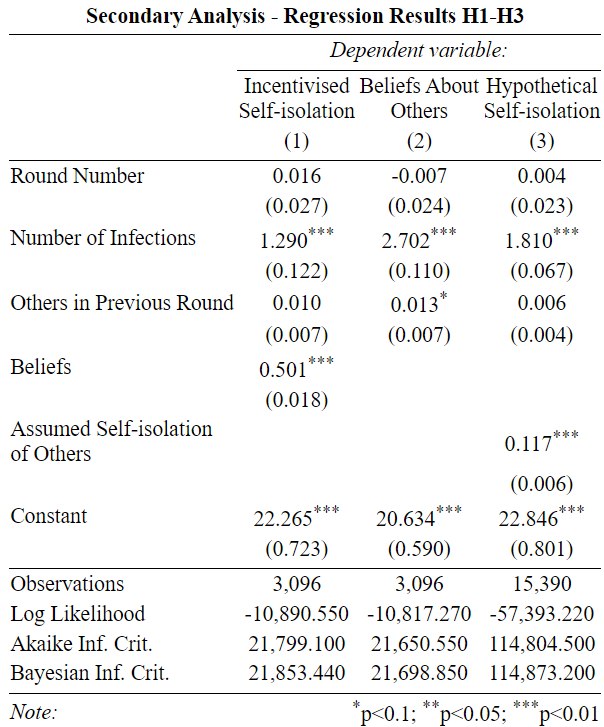

Supplement: Supplementary file 1 — Supplementary Information. [file 41598_2022_17642_MOESM1_ESM.docx]
